# Origin, distribution, and function of three frequent coding polymorphisms in the gene for the human P2X7 ion channel

**DOI:** 10.3389/fphar.2022.1033135

**Published:** 2022-11-18

**Authors:** Waldemar Schäfer, Tobias Stähler, Carolina Pinto Espinoza, Welbeck Danquah, Jan Hendrik Knop, Björn Rissiek, Friedrich Haag, Friedrich Koch-Nolte

**Affiliations:** ^1^ Institute of Immunology, University Medical Center Hamburg-Eppendorf, Hamburg, Germany; ^2^ Department of Neurology, University Medical Center Hamburg-Eppendorf, Hamburg, Germany

**Keywords:** ATP, adenosine triphosphate, P2X7, ATP-gated P2X ion channel, ion channel, purinergic receptor, single nucleotide polymorphism, loss of function

## Abstract

P2X7, an ion channel gated by extracellular ATP, is widely expressed on the plasma membrane of immune cells and plays important roles in inflammation and apoptosis. Several single nucleotide polymorphisms have been identified in the human *P2RX7* gene. In contrast to other members of the P2X family, non-synonymous polymorphisms in P2X7 are common. Three of these occur at overall frequencies of more than 25% and affect residues in the extracellular “head”-domain of P2X7 (155 Y/H), its “lower body” (270 R/H), and its “tail” in the second transmembrane domain (348 T/A). Comparison of the P2X7 orthologues of human and other great apes indicates that the ancestral allele is Y—R—T (at 155–270–348). Interestingly, each single amino acid variant displays lower ATP-sensitivity than the ancestral allele. The originally published reference sequence of human P2X7, often referred to as “wildtype,” differs from the ancestral allele at all three positions, i.e. H—H—A. The 1,000 Genome Project determined the sequences of both alleles of 2,500 human individuals, including roughly 500 persons from each of the five major continental regions. This rich resource shows that the ancestral alleles Y155, R270, and T348 occur in all analyzed human populations, albeit at strikingly different frequencies in various subpopulations (e.g., 25%–59% for Y155, 59%–77% for R270, and 13%–47% for T348). BLAST analyses of ancient human genome sequences uncovered several homozygous carriers of variant P2X7 alleles, possibly reflecting a high degree of inbreeding, e.g., H—R—T for a 50.000 year old Neanderthal, H—R—A for a 24.000 year old Siberian, and Y—R—A for a 7,000 year old mesolithic European. In contrast, most present-day individuals co-express two copies of P2X7 that differ in one or more amino acids at positions 155, 270, and 348. Our results improve the understanding of how P2X7 structure affects its function and suggest the importance of considering P2X7 variants of participants when designing clinical trials targeting P2X7.

## Introduction

Extracellular ATP is an important signaling molecule that can regulate numerous biological processes (Zimmermann 2000; la Sala et al., 2003; Lazarowski et al., 2003; Burnstock 2006; Khakh and North, 2006). The human genome encodes seven ionotropic P2X purinoceptors, i.e., ATP-gated ion channels designated P2X1-P2X7 (with corresponding genes designated *P2RX1-P2RX7*) (Di Virgilio et al., 2001; North 2002; Vial et al., 2004). Among these, P2X7 is widely expressed on the plasma membrane of immune cells and plays important roles in inflammation and apoptosis (Di Virgilio 1995; Surprenant et al., 1996; Ferrari et al., 2006). P2X7 differs from other P2X receptors by its relatively low sensitivity to ATP and by its relatively long cytoplasmic C-terminus (North and Surprenant, 2000; North, 2002). P2X7 has been proposed to function as a key regulator of inflammation and plays a crucial role in the ATP-dependent processing and release of the leader-less proinflammatory cytokines IL-1β and IL-18 (Ferrari et al., 1997; Perregaux et al., 2000; MacKenzie et al., 2001; Solle et al., 2001; Gudipaty et al., 2003; Mariathasan et al., 2006). P2X7 has been implicated in the killing of mycobacteria and chlamydia residing inside macrophages (Lammas et al., 1997; Fairbairn et al., 2001; Coutinho-Silva et al., 2003), fusion of monocyte-derived cells into multinucleated epithelioid cells (Chiozzi et al., 1997; Di Virgilio et al., 1999), the apoptosis of regulatory T cells (Seman et al., 2003; Aswad et al., 2005; Auger et al., 2005; Hubert et al., 2010), and the shedding of the CD62L homing receptor from circulating T cells (Jamieson et al., 1996; Gu et al., 1998; Seman et al., 2003).

The crystal structure of truncated panda (*Ailuropoda melanoleuca*) P2X7 encompassing the extracellular ATP-binding domain and two transmembrane segments (Karasawa and Kawate, 2016; Karasawa et al., 2017) as well as the cryo-EM structure of native rat P2X7 (McCarthy et al., 2019) have provided insights into the modes of ATP binding and gating.

In the human population, single amino acid polymorphic substitutions (R307Q, T357S, E496A, and I568N) and a 5′-intronic splice site polymorphism have been shown to result in a reduced or absent P2X7 functions (Gu et al., 2001; Wiley et al., 2003; Gu et al., 2004; Sluyter et al., 2004; Skarratt et al., 2005; Shemon et al., 2006). Moreover, splice variants leading to receptors lacking the cytoplasmic tail have been detected, which may account for reduced P2X7 functions in some normal tissues as well as in tumor cells overexpressing those variants by antagonizing the function of the normal variant (Feng et al., 2006). Two polymorphisms in the sequence of the human P2X7, H155Y, and A348T were reported as gain-of-function mutants (Cabrini et al., 2005).

The 1,000 Genomes Project provides the reconstructed genomes of a global sample of 2.504 individuals from 26 populations with about 500 samples from each of five continent ancestry groups in Africa (AFR), East Asia (EAS), Europe (EUR), South Asia (SAS), and the Americas (AMR) (Clarke et al., 2012; Delaneau et al., 2014; Auton et al., 2015; Clarke et al., 2017). This resource provides a rich data set on the distribution of common and rare genetic variations in humans. A typical human genome contains an estimated 4.1-5 million single nucleotide polymorphisms (SNPs) and short indels and 2,100 to 2,500 structural variants that affect more bases, e.g., large deletions, copy number variants, inversions (Auton et al., 2015). The majority of SNPs are rare (64 Mio with a frequency of <0.5%), a smaller proportion (12 Mio) has a frequency between 0.5% and 5% and even fewer SNPs (8 Mio) have a frequency of >5%. A typical human genome contains 10,000 to 12,000 sites with peptide-altering sequence variants, i.e., on the average one such variant in every other protein-coding gene.

The goal of this study was to compare the distribution of coding SNPs in human P2X7 to those in its paralogues, its orthologues in today’s great apes and DNA samples from ancient humans. We established protocols to determine the alleles of P2X7 and measure their sensitivity to ATP in human blood samples. Our results indicate that most humans co-express two alleles of P2X7 that differ in one or more amino acids. This correlates with high interindividual variation in the sensitivity of lymphocytes to extracellular ATP.

## Methods

### Database searches and sequence analyses

Data on coding SNPs in the *P2RX* gene family was retrieved from the 1,000 Genome Project *via* the Ensemble genome browser (Cunningham et al., 2022). Blastp and Tblastn (Madden et al., 1996; Ye et al., 2006) searches of the NCBI nucleotide database were performed using the “wildtype” human P2X7 sequence (Rassendren et al., 1997) as query. P2X7 amino acid sequences were aligned using T-Coffee (Notredame et al., 2000).

### Sequencing of human *P2RX7* gene fragments and structural modelling of P2X7 variants

Genomic DNA was isolated from peripheral blood mononuclear cells (PBMC) using a commercial DNA isolation kit (StemCell Technologies). Appropriate primer pairs were used to PCR amplify exons 5, 8, and 11. PCR amplification products were analyzed by agarose gel electrophoresis. Bands of the expected size were extracted from gel slices using a commercial DNA fragment purification kit (Qiagen). Fragments were sequenced using appropriate primers. DNA sequences were analyzed using Genescript (Hudek et al., 2003). P2X7 variants were modelled using AlphaFold 2 (Jumper et al., 2021; Jumper and Hassabis, 2022).

### Evaluation of the functionality of P2X7 variants by flow cytometry

PBMC were pre-incubated with P2X7-specific mAb L4 (Buell et al., 1998) or Nb Dano1 (Danquah et al., 2016) for 15 min at 4°C and then further incubated for 20 min at 37°C in RPMI medium in the absence or presence of ATP (Seman et al., 2003; Adriouch et al., 2008). Cells were then stained with fluorochrome-conjugated Annexin V and antibodies against CD62L (DREG-56), CD4 (RPA-T4), and CD8 (SK1) for 20 min before analysis by flow cytometry on a FACS-Canto (BD).

## Results

### The human *P2RX7* gene contains sixteen coding SNPs with a minor allele frequency of >0.5%

We used the resource of the 1,000 Genome Project (Clarke et al., 2012; Delaneau et al., 2014; Auton et al., 2015; Clarke et al., 2017) to determine the distribution of common coding mutations in *P2RX7*. The results reveal three frequent variants (minor allele frequency >25%) (Table 1). Two of these affect amino acid residues in the extracellular ATP-binding domain (Y155H, R270H) while the third affects an amino acid in the transmembrane domain (T348A). The *P2RX7* gene contains four further common non-synonymous variants with a minor allele frequency of >5% (E496A, T357S, Q460R, and V76A) and nine coding SNPs with a frequency of 0.5%–5%. Table 1 also shows the minor allele frequencies of eleven coding SNPs determined previously by the Wiley lab by genotyping *P2RX7* of 3,430 Caucasians (Stokes et al., 2010). Three of these coding SNPs occur at more than two-fold higher frequency in the Caucasian population.

**TABLE 1:** Frequencies of 20 coding SNPs in the human P2X7 gene. SNPs are sorted according to the minor allele frequency (maf) in the sample of the 1,000 Genomes Project; maf (Stokes) refers to a sample of 3,430 Caucasians genotyped by Stokes et al., 2010.

SNP	maf	maf (Stokes)	residue	exon	mutation
rs208294	0.461	0.439	155	5	Y/H
rs1718119	0.308	0.400	348	11	A/T
rs7958311	0.289	0.255	270	8	R/H
rs3751143	0.192	0.175	496	13	E/A
rs2230911	0.144	0.083	357	8	T/S
rs2230912	0.068	0.170	460	13	Q/R
rs17525809	0.054	0.062	76	2	V/A
rs2230913	0.037		521	13	H/Q
rs10160951	0.036		430	12	P/R
rs28360459	0.029		433	13	A/V
rs74357548	0.011		423	12	D/N
rs28360447	0.010	0.018	150	5	G/R
rs7958316	0.010	0.020	276	8	R/H
rs1653624	0.009	0.029	568	13	N/I
rs34219304	0.010		522	13	V/I
rs16950860	0.008		270	8	R/C
rs28360457	0.004	0.013	307		R/Q
rs28360460	0.003		578		R/Q
rs28360445	0.002		117		R/W
rs200840067	0.002		582		P/S

### The six paralogues of the *P2RX7* gene together contain seven SNPs with a minor allele frequency of >0.5%

**FIGURE 1:**
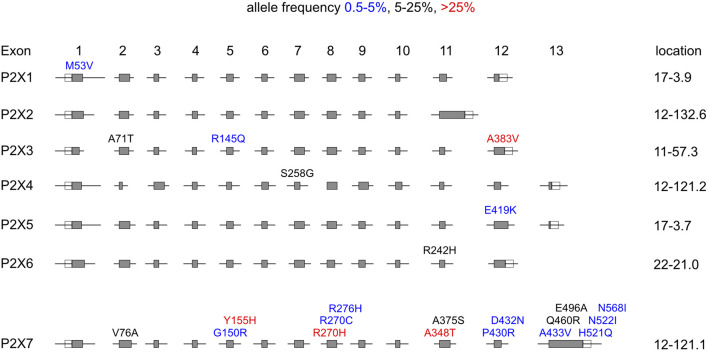
Distribution of coding SNPs in members of the human *P2RX* gene family. Schematic illustration of the exon/intron structures of the human *P2RX* gene family. The positions of coding SNPs are indicated above the exons. The color of the font indicates the frequency of the respective mutation in the overall human population: >25% red, 5%–20% black, 0.5%–5% blue among the ∼2.500 individuals from five continental regions analyzed by the 1,000 Genomes Project. The amino acid residues of the major and minor alleles are given in single letter code, before and after the position of the amino acid residue in the native protein, respectively. Numbers on the right indicate the chromosomal localization of the respective gene. *P2RX4* and *P2RX7* are immediate chromosomal neighbors on chromosome 12. *P2RX1* and *P2RX5* are separated by five other genes on Chromosome 17.

We performed similar analyses for the other six members of the *P2RX* gene family. The results reveal that the six paralogues of *P2RX7* contain much fewer coding SNPs than *P2RX7* (Table 2). *P2RX3* contains a single coding SNP with a frequency of >25% (A383E). *P2RX4* and *P2RX6* each contain a single coding SNP with a frequency of 5%–25% (S258G, R242H, respectively). And the six genes together contain only four further non-synonymous variants with a frequency of 0.5%–5%. Figure 1 schematically illustrates the location of the coding SNPs with frequencies >0.5% in the human *P2RX* gene family.

**TABLE 2:** Coding SNPs in other members of the P2RX gene family with a frequency >0.5%. nd = none detected.

SNP	Gene	exons	Length (aa)	maf	exon	mutation
rs34617528	*P2RX1*	12	399	0.008	1	M53V
Nd	*P2RX2*	11	471	nd	nd	nd
rs34572680	*P2RX3*	12	397	0.019	2	A71T
rs115850675	*P2RX3*	12	397	0.005	5	R145Q
rs2276038	*P2RX3*	12	397	0.432	12	A383V
rs25644	*P2RX4*	12	404	0.176	7	S258G
rs61748727	*P2RX5*	12	444	0.018	12	E419K
rs2277838	*P2RX6*	12	441	0.078	7	R242H

### The three high frequency polymorphisms of human P2X7 (H155Y, R270H, and A348T) occur in all major human subpopulations

**FIGURE 2:**
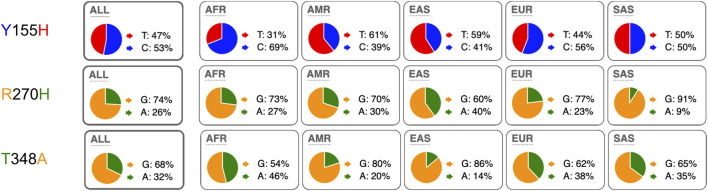
Distribution of minor and major alleles of the three most frequent coding SNPs in the human P2X7 gene (coding for amino acids 155, 270, and 348). The pie charts illustrate the allele frequencies of the major and minor alleles of P2RX7 encoding amino acid positions 155, 270, and 348 of human P2X7 in humans from the five major continental regions. The data were retrieved from the 1,000 Genomes Project. ALL: (*n* = 2.504 individuals) with about 500 from each region: AFR: African, AMR: American, ESN: East-Asian, SAS South-Asian, EUR: European. The nucleotide of the respective alleles is indicated next to the pie chart, the encoded amino acid residues is indicated on the left using the same color coding as in the pie charts. AFR: African (*n* = 247), AMR: American (*n* = 181), ASN: Asian (*n* = 286), EUR: European (*n* = 379). ALL: (*n* = 1.092 humans).

We next compared the allele frequencies of the three most frequent coding SNPs in human *P2RX7* in different human populations. The results show that both alleles are found in all analyzed human populations, albeit with strikingly different allele frequencies in some subpopulations (Figure 2). For example, 86% of alleles of Peruvians in Lima (PEL) carry the SNP coding for Histidine at position 155, whereas 78% of alleles of Luhya in Webuye, Kenya (LWK) encode Tyrosine at this position. For the 270 R/H SNP, the majority of alleles in all human populations encode R270 (from as low as 56% of Chinese in Beijing (CHB) to as high as 94% of Indian Telugu in the UK (ITU). And for the SNP encoding residue 348, the vast majority of alleles (94%) in Peruvians in Lima (PEL) encode A348, while a small majority of alleles of Esan in Nigeria (ESN) (53%) encode T at this position.

### Y155, R270, and T348 represent the ancestral haplotype of P2X7

The cDNA for human P2X7 that was originally cloned by Rassendren et al. (1997) from a commercially available cDNA library (Clontech, San Diego, United States) contained H155, H270 and A348, i.e., it contained the major allele of the AMR population at positions 155 and 348, but the minor allele at position 270. In this paper, Rassendren et al. (1997) compared the predicted amino acid sequence of human P2X7 with that of its previously cloned orthologue from rat (Surprenant et al., 1996). Interestingly, at each of these positions rat P2X7 carries the respective other allele (Y155, R270, and T348) (Surprenant et al., 1996).

**FIGURE 3:**
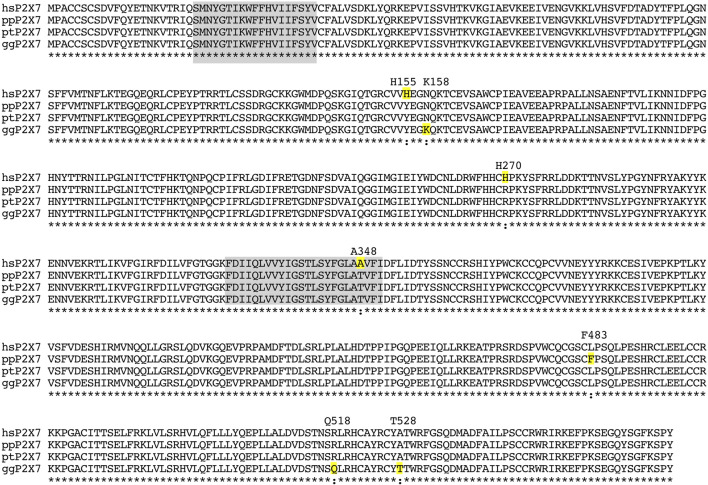
Amino acid sequence alignment of the P2X7 orthologues of the great apes. Residues highlighted in grey correspond to the two transmembrane domains. Residues highlighted in yellow indicate substitutions relative to the presumed ancestral version. The sequence of chimpanzee P2X7 corresponds completely to the presumed ancestral allele. Amino acid residues that deviate from the ancestral allele are indicated with their respective positions in the native protein above the alignment. hs: *homo sapiens*; pp: *pan paniscus* (bonobo); pt: *pan troglodytes* (chimpanzee); gg: gorilla gorilla.

Using the “wildtype” sequence cloned by Rassendren et al. (1997) as query for BLAST analyses of the NCBI gene database, we obtained the deduced amino acid sequences of the P2X7 orthologues from chimpanzee, bonobo, and gorilla. Figure 3 shows the amino acid sequence alignment of the P2X7 orthologues of the great apes. The results show that all sequenced great apes carry Y155, R270, and T348, indicating that these represent the ancestral haplotype of P2X7. The “wildtype” human P2X7 deviates from the chimpanzee sequence only at these positions (H155, A270, and A348). The bonobo P2X7 sequence differs from human and the other great apes only at position F483L. Gorilla P2X7 differs from that of the other great apes at three other positions (N158K, R518Q, and A528T).

### Mutations from the ancestral allele were present already in ancient humans

With BLAST analyses we also retrieved P2X7 variants from several ancient human DNA samples (Table 3). Interestingly, a 7.000 year old Spanish human carried the ancestral Y155 allele, while the older Siberian (24 ka) and Neanderthal (60 ka) carried the variant H155 allele. At position 348, the Neanderthal carried the ancestral T348 allele, whereas the younger Siberian and Spanish humans carried the variant A348 allele. The results indicate that both, the ancestral and variant alleles, were present in ancient human DNAs.

**TABLE 3:** Distribution of three most common human P2X7 coding SNPs in ancient humans.

residue	Ancestral	“wt"”	60 ka Neanderthal	24 ka Siberian	7 ka Spanish
155	Y	H	H	H	Y
348	R	H	R	R	R
270	T	A	T	A	A

### Most present-day humans carry two different alleles of *P2RX7*

**FIGURE 4:**
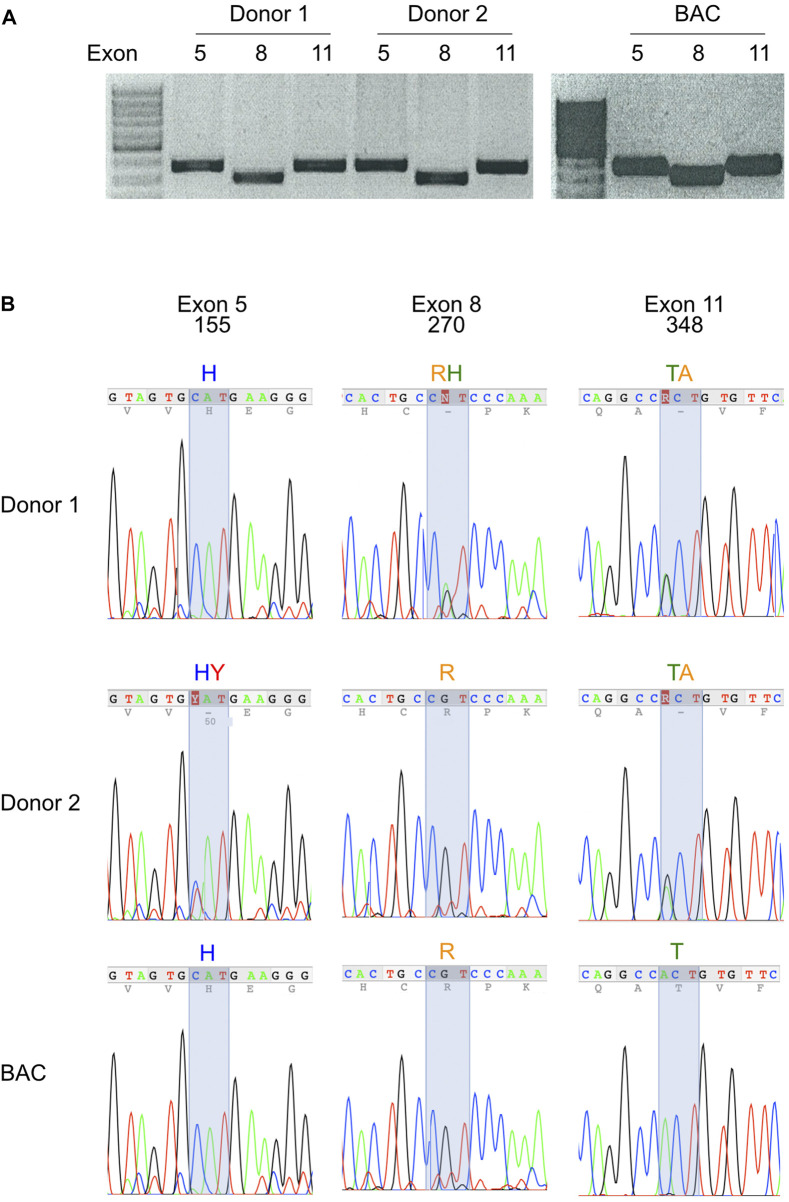
Most modern-day humans carry two variants of the P2X7 gene. **(A)** Exons 5, 8, and 11 of the human P2X7 gene were PCR-amplified from genomic DNA of peripheral blood leukocytes. Amplification products were size fractionated by agarose gel electrophoresis and stained with ethidium bromide. **(B)** PCR amplification products were purified from gel fragments and sequenced with internal primers. Fluorograms illustrate homozygosity or heterozygosity of PCR amplification products at the positions of the three most frequent SNPs of human *P2RX7*. The deduced amino acids are indicated above the fluorographs.

The allele frequencies illustrated by the pie charts in Figure 2 suggest that most human beings carry two different alleles of P2X7. Native P2X7 is expressed as a homotrimer on the cell surface. If both alleles were co-expressed, P2X7 receptors on the cell surface would be composed of a mixture of four different combinations of two polypeptide chains. In order to identify the variants of P2X7 at these positions in different human samples, we established a protocol to PCR amplify and sequence the three exons (5, 8, and 11) carrying these mutations. The results show that this strategy allows the rapid genotyping of the P2XR7 gene in different individuals (Figure 4).

**FIGURE 5:**
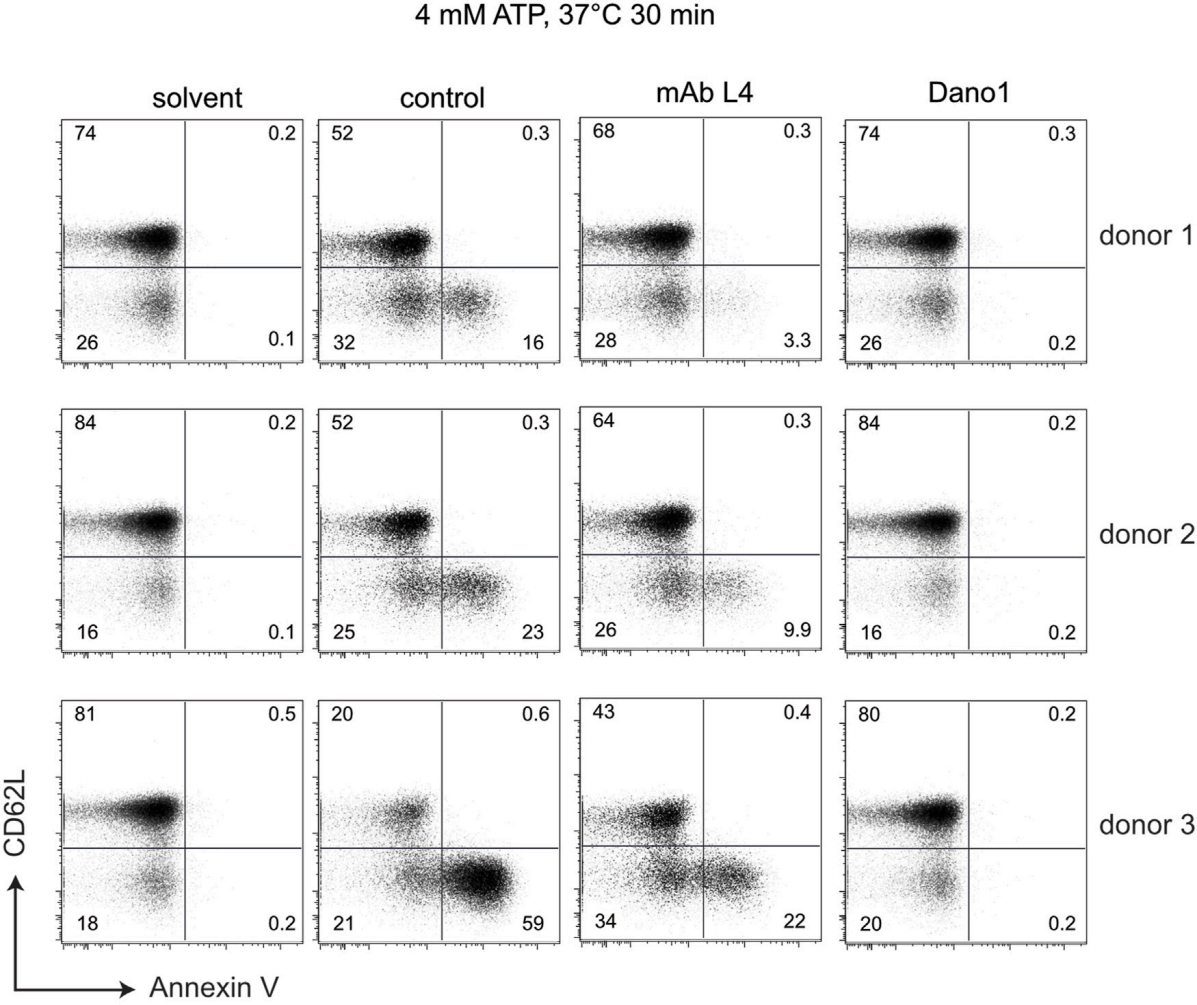
Sensitivity of peripheral blood T cells to ATP-induced shedding of CD62L varies with the genotype at positions 155, 270, and 348 of P2X7. Human blood samples were incubated for 30 min at 37°C in the absence (solvent) or presence of 4 mM ATP and the additional absence (control) or presence of the P2X7-antagonizing monoclonal antibody L4 or the nanobody Dano1. Cells were then washed and co-stained with antibodies directed against CD4 and CD62L as well as with Annexin V to visualize externalization of phosphatidylserine).

To determine whether the different genotypes affect the function of P2X7, we used an established, simple flow-cytometry assay (Danquah et al., 2016). For this, we treated BMCs for 30 min with ATP and monitored ATP-induced shedding of CD62L and externalization of phosphatidylserine by T cells (Figure 5). As a specificity control, we used P2X7-blocking antibodies, i.e., the nanobody Dano1 and the monoclonal antibody L4. The results show that individuals carrying the Y155 and/or T348 variants respond more sensitively to ATP than individuals carrying the H155 or A348 variants.

### The three high frequency SNPs code for amino acids in the head, body and tail domains of P2X7

**FIGURE 6:**
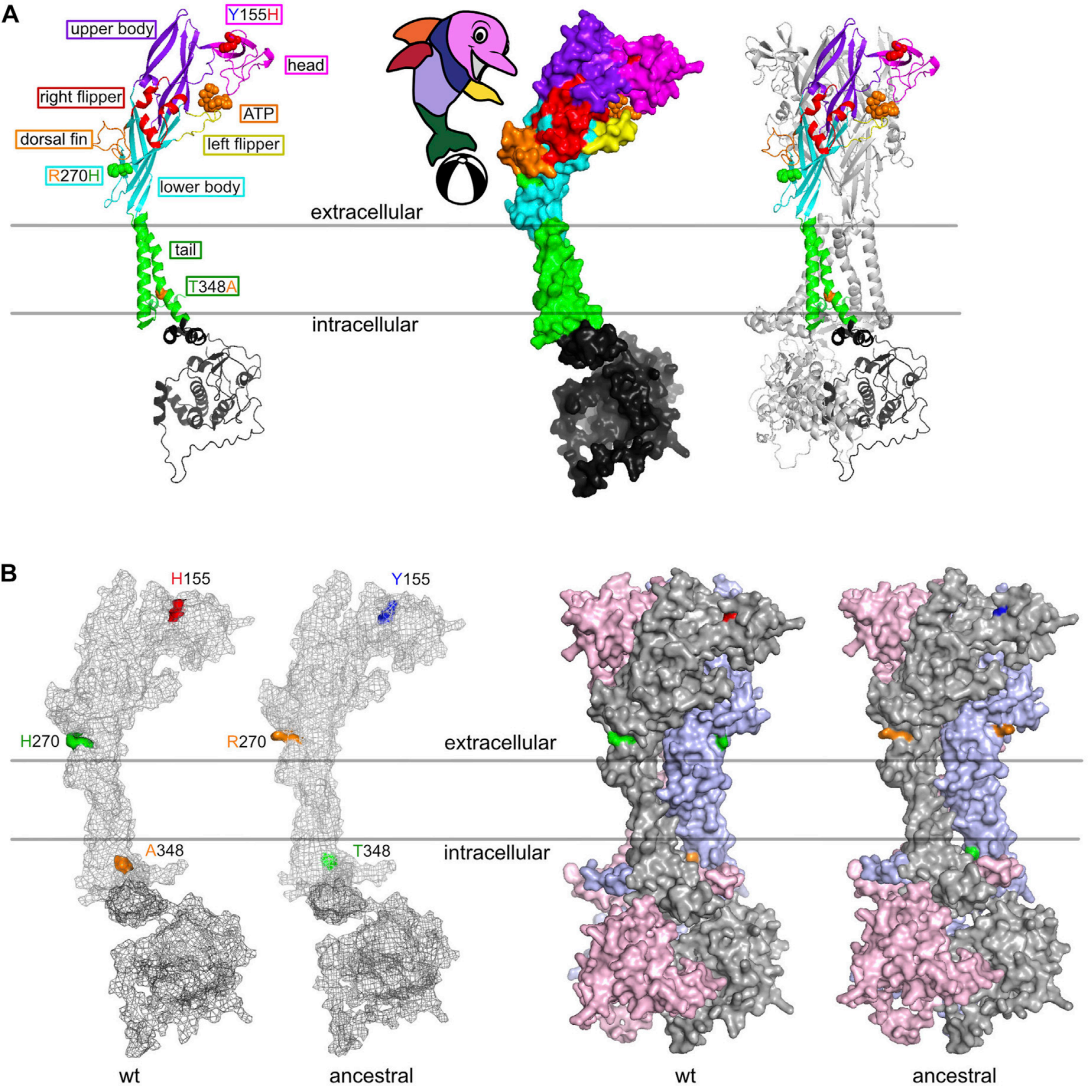
3D structure models of “wildtype” (HHA) and ancestral (YRT) human P2X7. **(A)** The ribbon diagram highlights the positions of the three frequent coding SNPs in the different “body-parts” of the dolphin model of P2X7 (Karasawa and Kawate, 2016). Y155H is located in the head domain, R270H in the right flipper, and A348T in the tail of the dolphin. **(B)** 3D-structure models were generated using AlphaFold 2 and color-coded in pymol. The three divergent amino acid residues are color-coded as in Figures 2, 4.

We used AlphaFold 2 to predict the 3D-structures of ancestral human P2X7 (Y155, R270, and T348) and “wildtype” P2X7 (H155, H270, and A348) (Figure 6). The cartoon model in Figure 6A illustrates the localization of these variants in the so-called dolphin model of P2X7. H155 is located in the extracellular head domain, H270R in the extracellular right flipper, and A348T in the second transmembrane domain, i.e. in the tail of the dolphin. Figure 6B shows the 3D models of the variant and wildtype P2X7 variants predicted by AlphaFold 2. The three variant residues are indicated in the color coding used also in Figure 2. The results indicate that the conformational changes induced by these substitutions are rather subtle.

## Discussion

Our results show that three coding SNPs of P2X7 occur at allele frequencies of more than 25%: 155 Y/H, 270 R/H, and 348 T/A. Furthermore, *P2RX7* contains 13 additional SNPs with a frequency of >0.5% (Table 1). This is in striking contrast to other members of the P2X gene family (Table 2): Only *P2RX3* contains a coding SNP with a frequency of >25% (A383E). *P2RX4*, the immediate chromosomal neighbour of *P2RX7* contains a SNP with a frequency of 18% (S258G) but no other SNPs with a frequency of >0.5%.

The three high frequency variant residues of *P2RX7* are located in the head, lower body, and tail domains of P2X7 (Figure 6). Comparison of the P2X7 orthologues of human and other great apes indicates that the ancestral allele is Y - R - T at these positions (Figure 4). The originally published reference sequence of human P2X7 is often referred to as “wildtype” (Rassendren et al., 1997). This variant H - H - A differs from the ancestral allele at all three positions. Analysis of the data of the 1,000 Genome Project reveals a broad range of frequencies of the ancestral P2X7 alleles at these positions in different human populations (e.g., 39%–69% for Y155, 60%–91% for R270, and 14%–46% for T348) (Figure 2).

In a seminal study, Stokes et al. (2010) genotyped the *P2RX7* gene of 3,430 Caucasian subjects and determined the minor allele frequencies for eleven coding SNPs (Table 1). As in previous studies (Rassendren et al., 1997), Stokes et al. (2010) refer to the common haplotype of Caucasians H155-H270-A348 as “wild-type” (P2X7-1). The authors performed detailed comparative analyses of P2X7-1 with a variant, designated P2X7-4, that corresponds to the ancestral Y155-R270-T348. Remarkably, however, the variant analyzed by Stokes et al. (2010) also carries the minor allele of the Q460R SNP (R460 instead of the ancestral Q460). Starting from “wildtype” P2X7-1, i.e., H155-H270-A348, Stokes et al. (2010) individually mutated residues 155 to Y and 270 to R, as well as 460 to R. Starting from P2X7-4, i.e., ancestral Y155-R270-T348 with the minor R460 allele, Stokes et al. (2010) individually mutated the “gain of function” residues back to wild type, i.e., Y155 to H, R270 to H, and T348 to A. By expressing these P2X7 variants in transiently transfected HEK cells, the authors verified gain of function effects of Y155, R270, and A348, as well as slight loss of function effect of R460. For the Q460R SNP, Stokes et al. (2010) noted a strong pairwise linkage disequilibrium with the three “gain of function” SNPs (Y155, R270, and T348), suggesting that the Q460R SNP was acquired by an individual carrying the ancestral P2X7 haplotype.

BLAST analyses of ancient human genome sequences uncovered several homozygous carriers of variant P2X7 alleles, possibly reflecting a high degree of inbreeding, e.g., H—R—T for a 50.000-year-old Neanderthal, H—R—A for a 24.000 year old Siberian, and Y—R—A for a 7.000 year old mesolithic European (Table 3). In contrast, most present-day human individuals co-express two copies of P2X7 that differ in one or more amino acids at positions 155, 270, and 348 (Figure 4). These results indicated an ancient origin of these alleles and the maintenance of a balanced polymorphism in most present-day human populations.

Peripheral blood T cells from individuals carrying the variant amino acid His at position 155 or Ala at position 348 show a reduced sensitivity to ATP-induced shedding of CD62L and externalization of phosphatidylserine compared to individuals that are homozygous for the ancestral variants Tyr and Thr at these positions (Figure 5). The fact that these ATP-induced effects are completely blocked by the highly specific P2X7-antagonizing Nanobody Dano1 (Figure 5) confirms that these are mediated through P2X7.

The major finding of our results is the strikingly high degree of allelic polymorphism in the P2XR7 gene in the human population. In fact, the data indicate that most human beings today are heterozygous at this locus and express two variants of the P2X7 protein that differ at one or more amino acid residues. It is difficult to determine whether the reduced function of the H155, H270, and A348 variants confer any benefit against inflammatory disease and/or enhanced susceptibility to infection, e.g., by Mycobacterium tuberculosis. The extremely high polymorphism at the MHC locus is thought to confer disease resistance on the population level, as a large number of variants in a population could provide a kind of group protection that checks the spread of infection (Trowsdale, 2011). By analogy, it is tempting to speculate that different sensitivities of human leucocytes in the population might be beneficial, e.g., by providing flexible response strategies to pandemic infectious agents on a population level. As P2X7 plays a key role in the activation of the inflammasome and in the release of IL-1β (Ferrari et al., 1997; Gudipaty et al., 2003; Ferrari et al., 2006; Mariathasans et al., 2006; Pelegrin and Surprenant, 2006), its sensitivity to ATP may have been moulded during evolution to prevent its excessive activation. Fine-tuning the sensitivity of P2X7-expressing inflammatory cells to very high levels of ATP released from cells at sites of tissue damage may have reduced the risk of exaggerated inflammatory responses. Conceivably, such evolutionary pressures may also be responsible for the numerous natural loss of function mutations found for human P2X7 and the remarkably high frequency of these mutations in human populations (Smart et al., 2003; Gu et al., 2004; Shemon et al., 2006).

P2X7 has been proposed as a therapeutic target for a variety of infectious (Di Virgilio et al., 2017), inflammatory (Arulkumaran et al., 2011; Adinolfi et al., 2018; Burnstock and Knight, 2018; Magalhaes and Castelucci, 2021; Verma et al., 2022), autoimmune (Cao et al., 2019), bone (Zeng et al., 2019; Dong et al., 2020), muscular (Gorecki, 2019), cardiovascular (Chen et al., 2018; Cisneros-Mejorado et al., 2020; Shokoples et al., 2021), oncological (Burnstock and Knight, 2018; Li et al., 2020; Drill et al., 2021; Pegoraro and Adinolfi, 2021; Zhu et al., 2021) and neurologic diseases (Rech et al., 2016; Bhattacharya, 2018, Domercq and Matut,e 2019, Engel et al., 2021), including COVID-19 (Pacheco and Faria, 2021), rheumatoid arthritis (Baroja-Mazo and Pelegrin, 2012; McInnes et al., 2014), diabetic retinopathy (Tassetto et al., 2021), alcoholic liver disease (Le Dare et al., 2021), Alzheimer (Illes et al., 2019) and depression (Deussing and Arzt, 2018; Wei et al., 2018; Huang and Tan, 2021), multiple sclerosis (Domercq and Matute, 2019), amyotrophic lateral sclerosis (Sluyter et al., 2017; McKenzie et al., 2022). Our results indicate that it may be wise to monitor the genotype of *P2RX7* and the ATP-sensitivity of its gene product P2X7 in clinical studies designed to evaluate the therapeutic benefit of P2X7-targeting drugs and antibodies (Bartlett et al., 2014; De Marchi et al., 2016; Park and Kim, 2017; Young and Gorecki, 2018; Koch-Nolte et al., 2019; Recourt et al., 2020; Mishra et al., 2021; Genetzakis et al., 2022). The assays shown in Figures 4, 5 provide a straightforward protocol to do so.

Considering that P2X7 is a key player in inflammation, selection in the human population of numerous variants displaying reduced function may confer some benefit against the deleterious effects of excessive inflammation. The very low sensitivity of P2X7 to extracellular ATP, as compared to the other P2X receptor, may in itself also reflect the action of selective pressures acting to lower the level of inflammation. Our results may aid in understanding the relation between P2X7 structure and function as well as the evolutionary pressures that maintain its low sensitivity to ATP.

## Data Availability

The original contributions presented in the study are included in the article, further inquiries can be directed to the corresponding author.

## Abbreviations

ATP, adenosine triphosphate; maf, minor allele frequency; P2X7 ATP-gated P2X ion channel, ionotropic purinergic receptor; *P2RX7*, gene encoding P2X7; SNP, single nucleotide polymorphism.
